# Healthy and diabetic primary human osteoblasts exhibit varying phenotypic profiles in high and low glucose environments on 3D-printed titanium surfaces

**DOI:** 10.3389/fendo.2024.1346094

**Published:** 2024-07-03

**Authors:** Nicholas Allen, Alexandra Hunter Aitchison, Bijan Abar, Julian Burbano, Mark Montgomery, Lindsey Droz, Richard Danilkowicz, Samuel Adams

**Affiliations:** Duke University Medical Center, Duke University, Durham, NC, United States

**Keywords:** diabetes, Charcot, 3D printing, osteointegration, implants, additive manufacturing, BGLAP, Bmp7

## Abstract

**Background:**

The revolution of orthopedic implant manufacturing is being driven by 3D printing of titanium implants for large bony defects such as those caused by diabetic Charcot arthropathy. Unlike traditional subtractive manufacturing of orthopedic implants, 3D printing fuses titanium powder layer-by-layer, creating a unique surface roughness that could potentially enhance osseointegration. However, the metabolic impairments caused by diabetes, including negative alterations of bone metabolism, can lead to nonunion and decreased osseointegration with traditionally manufactured orthopedic implants. This study aimed to characterize the response of both healthy and diabetic primary human osteoblasts cultured on a medical-grade 3D-printed titanium surface under high and low glucose conditions.

**Methods:**

Bone samples were obtained from six patients, three with Type 2 Diabetes Mellitus and three without. Primary osteoblasts were isolated and cultured on 3D-printed titanium discs in high (4.5 g/L D-glucose) and low glucose (1 g/L D-Glucose) media. Cellular morphology, matrix deposition, and mineralization were assessed using scanning electron microscopy and alizarin red staining. Alkaline phosphatase activity and L-lactate concentration was measured *in vitro* to assess functional osteoblastic activity and cellular metabolism. Osteogenic gene expression of *BGLAP*, *COL1A1*, and *BMP7* was analyzed using reverse-transcription quantitative polymerase chain reaction.

**Results:**

Diabetic osteoblasts were nonresponsive to variations in glucose levels compared to their healthy counterparts. Alkaline phosphatase activity, L-lactate production, mineral deposition, and osteogenic gene expression remained unchanged in diabetic osteoblasts under both glucose conditions. In contrast, healthy osteoblasts exhibited enhanced functional responsiveness in a high glucose environment and showed a significant increase in osteogenic gene expression of *BGLAP*, *COL1A1*, and *BMP7* (p<.05).

**Conclusion:**

Our findings suggest that diabetic osteoblasts exhibit impaired responsiveness to variations in glucose concentrations, emphasizing potential osteoblast dysfunction in diabetes. This could have implications for post-surgery glucose management strategies in patients with diabetes. Despite the potential benefits of 3D printing for orthopedic implants, particularly for diabetic Charcot collapse, our results call for further research to optimize these interventions for improved patient outcomes.

## Background

The technological revolution of 3D printing is making notable strides in various industries, including healthcare. Its application in orthopedics, specifically for creating custom implants for spine, trauma, and extremity surgeries, has shown tremendous promise (1–3). 3D printing offers an alternative to traditional manufacturing techniques by building components layer by layer, which can lead to improved osseointegration, a critical factor in successful implantation and healing (4–6). Among the 3D printed materials, medical grade titanium alloy (Ti6Al4V) has emerged as a favorable option for orthopedic applications due to its excellent strength-to-weight ratio and resistance to corrosion (7, 8). In addition, implants fabricated using medical grade titanium alloy have been extensively characterized for their degradation and biocompatibility, making them a popular choice in orthopedics (9). The layer-by-layer fusion of this manufacturing process results in a natural “as-printed” surface roughness not seen with traditional milled implants, which may promote better bone in-growth and osseointegration (10, 11). Consequently, 3D printed implants are finding increasing use in foot and ankle surgery to address large bone defects from diabetic Charcot collapse, a severe outcome of diabetic peripheral neuropathy (12–14).

With the advent of 3D printed implants in the orthopedic field, studies examining the osteogenic response to these materials have multiplied, but most research has focused on the response of healthy osteoblasts (15, 16). However, diabetes has a complex impact on bone metabolism, which often leads to nonunion and decreased osseointegration to traditionally manufactured orthopedic implants (17–20). In an animal model of diabetes, implantation of titanium screws into distal femurs of rats resulted in bony derangement, poor bone maturity and lower expression of osteogenic genes (21). Moreover, the use of implants in patients with diabetes is further complicated by the disease’s metabolic effects, which can impair bone healing and remodeling (22, 23). Despite these challenges, the response of diabetic osteoblasts to 3D printed surfaces in a clinical setting remains largely unclear.

In this study, we aimed to bridge this gap by characterizing the osteoblastic activity and functional phenotypes of both healthy and diabetic primary human osteoblasts cultured on a medical-grade 3D-printed titanium surface in the presence of high and low glucose environments. The hallmark of diabetes is elevated blood glucose. Therefore, diabetic osteoblasts are conditioned to an elevated glucose environment. However, after orthopedic surgery, an attempt is made to keep diabetic patients’ glucose concentrations in a normal range to decrease postoperative complications. It is unknown as to whether altering postoperative glucose available to diabetic osteoblasts, alters their function. Additionally, in order to characterize additional phenotypic changes that arise in the presence of an *in vitro* osteoblast-titanium surface environment, our study sought to investigate the variations in cell morphology, matrix deposition, and mineralization that play a prominent role osteoblast adhesion and integration into 3D fabricated titanium implant materials currently utilized in orthopedics.

Understanding the interaction of osteoblasts with 3D printed titanium implants in diabetic conditions is critical given the increasing prevalence of diabetes and the frequent need for orthopedic surgeries in this population (24, 25). We hypothesize that the disrupted glucose milieu in diabetes will affect osteoblast functionality on 3D printed discs compared to healthy osteoblasts when exposed to low and high glucose environments. To our knowledge, this is the first study to investigate the effects of varying glucose levels on diabetic osteoblasts interacting with 3D printed titanium. We believe that our findings will provide important insights to guide application of 3D printed orthopedic implants and postoperative glucose control, particularly for use in patients with diabetes.

## Methods

### Patient cohort

Following institutional review board approval, discarded bone samples were obtained from six patients at the time of their foot and ankle surgery performed by the senior author. Patient demographics can be seen in **Table 1**.

**Table 1:** Patient demographics at time of surgery and osteoblast collection.

	Healthy		Diabetic		
	Mean/%	SD	Mean/%	SD	P-Value
Age (years)	37.6	12.7	60.6	21.6	0.21
Sex (Female)	66.6%	0.6%	33.3%	0.6%	0.56
Blood glucose (mg/dL)	82.7	8.02	276.7	47.9	.002
Hemoglobin A1C	-	–	7.5%	0.4%	–

SD, Standard deviation.

**Figure 1:**
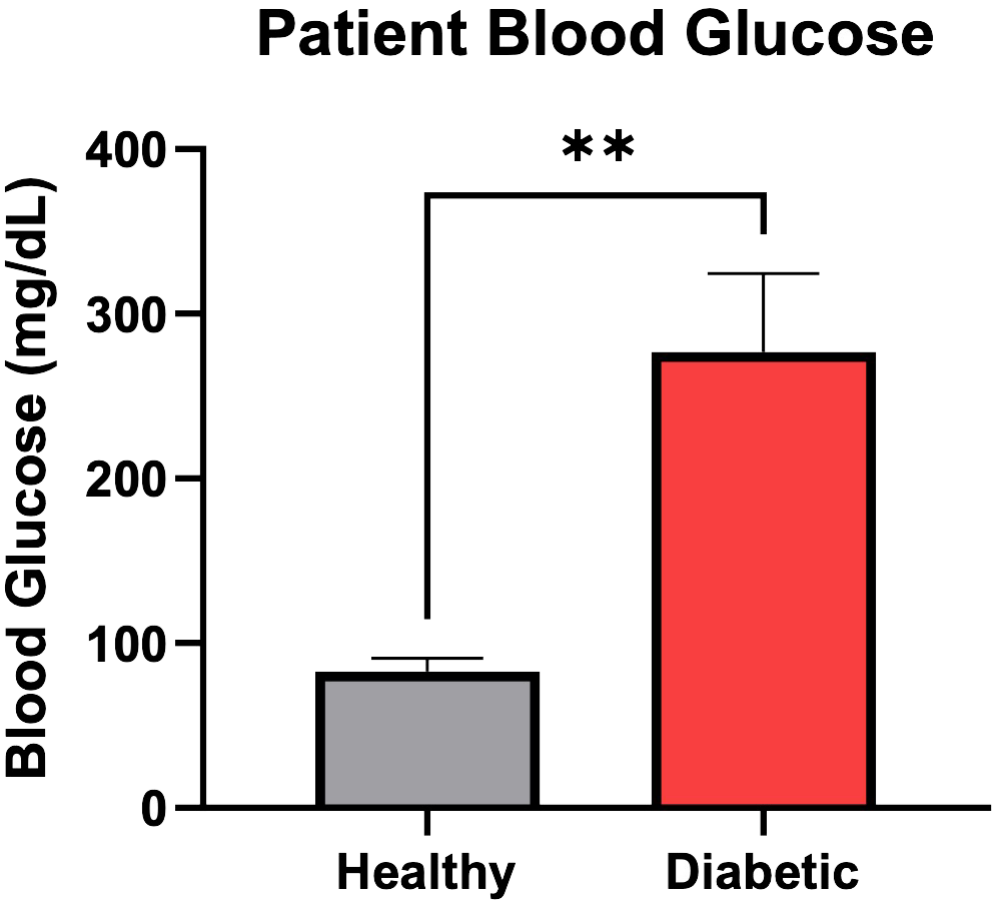
Patient blood glucose (mg/dL) concentration at time of surgery. Error bars represent SEM, **p<.01.

Three of these patients had a diagnosis of Type 2 Diabetes Mellitus, a mean age of 60.6 years and were 66.6% female. The other three patients had no current or historical diagnosis of diabetes or prediabetes, had a mean age of 37.6 years and were 66.6% male. The average patient blood glucose concentration at time of surgery in the diabetic osteoblast group was significantly higher than the healthy group (2.76 g/L vs 0.83 g/L, P=.002) (**Figure 1**). The average preoperative hemoglobin A1C level in the diabetic group was 7.5% ± 0.4%.

### Primary osteoblast isolation

Healthy (n=3) and diabetic (n=3) primary osteoblasts were isolated from discarded healthy and Charcot patient bone fragments according to methods established by Taylor et al. (26) Briefly, discarded bone fragments were collected at the time of surgery and processed in a biosafety cabinet to remove any soft connective tissue and coagulated marrow from the outer surface of the bone. Bone fragments were placed in a sterile petri dish containing 5-10 mL of phosphate buffered saline (PBS) containing 1% antibiotic-antimycotic (anti/anti) (Cat.#15240096, Gibco) and cut into 2-4 mm fragments. The fragments were transferred to into a 50 mL universal container with 10-15 mL PBS + anti/anti and vortexed 3 x 5 sec before decanting off the supernatant to remove hematopoietic marrow, marrow fat, and dislodged cells. This process was repeated for a total of 6 washes until the bone fragments assumed a white ivory-like appearance. Fragments were incubated in 1% trypsin for 10 min at 37°C, washed with Minimal Essential Medium (a-mem) (12571063, Gibco), then PBS. Fragments were allowed to incubate in 0.2% collagenase solution for 30 min at 37°C, washed with a-mem, and cultured at a density of 0.2-0.5 g tissue per 100 mm diameter in a cell culture dish containing a-mem, anti/anti, and 10% fetal bovine serum (FBS) (Cat.#16000044, Gibco) at 37°C/5% CO_2_. Outgrowing cells were expanded to confluence in expansion medium (a-mem, 10% FBS, 1% anti/anti) using standard tissue culture technique and pooled to generate respective healthy and diabetic osteoblast populations.

### 3D fabrication of Ti6Al4V discs

Both the discs used for cell culture and mechanical testing were manufactured via the Laser Powder Bed Fusion (L-PBF) technique using a 3D Systems ProX DMP320 system, as previously described by Abar et al. (10). Medical-grade titanium alloy powder (Ti6Al4V ELI), with a standard particle diameter of 35 μm, was utilized to meet ASTM F3001 standards, which are essential for implanted medical devices. The discs, printed with a diameter of 5 cm, featured an unpolished surface topography. The L-PBF process fuses spherical metal powder units, resulting in a distinctive rough topography on the surfaces of the printed discs. This surface roughness was intentionally preserved post-production.

### Characterization of Ti6Al4V discs

Surface roughness was quantified using an Olympus LEXT OLS4000 Confocal Microscope at 20x magnification, reporting roughness parameters Ra (average roughness) and Rz (maximum peak to valley height). Mechanical properties, including Ultimate Tensile Strength (UTS), Yield Strength, Young’s Modulus, and Fracture Strain, were assessed through tensile testing following ASTM E8M standards on a Test Resources TR830 axial testing frame. Yield Strength was specifically calculated using the 2% offset method, and sample diameters were verified with digital calipers prior to testing. Details of these characteristics are provided in **Table 2** (11).

**Table 2:** Surface roughness and mechanical properties of Ti6Al4V discs (11).

Ra (um)	Rz (um)	UTS (MPa)	Yeild Strength (MPa)	Young's Modulus (GPa)	Fracture Strain (%)
6.7 ± 1.1	33 ± 4.5	1053 ± 8.2	992.1 ± 8.1	103.3 ± 5.8	12.6 ± 1.3

Data represented as average ± standard deviation.

### Cell culture

The 3D printed discs were autoclaved at 250°F for 85 minutes before being placed in sterile 48 well plates. Healthy or diabetic osteoblasts were seeded at 10e^4^ cells/disc in the 48 well plates and allowed to adhere for 4 hours. The expansion media was aspirated, and the healthy and diabetic-laden discs were cultured in complete osteogenic medium (DMEM, 10% FBS, 1% anti/anti, 10 mM b-glycerophosphate, 50 ug/mL ascorbic acid) containing high (4.5 g/L D-glucose) or low glucose (1 g/L D-Glucose) concentrations. Samples were cultured to respective timepoints and harvested for analysis.

### Scanning electron microscopy

At days 14 and 28 discs were fixed in 10% neutral buffered formalin for scanning electron microscopy (SEM). Fixed samples were washed twice with 1× PBS and dehydrated with increasing ethanol concentrations and washed 3 times for 10 min each in respective concentrations (10, 30, 50, 70, 90, 100%). Samples were washed with Hexamethyldisilazane (Electron Microscopy Sciences) 3 times and allowed to incubate until reagent evaporated. Samples were sputter coated with gold for 400 s at 12 v (Denton Desk V) then imaged at 50× and 1,000× magnifications with a Tabletop Scanning Electron Microscope (Hitachi TM3030Plus).

### Alizarin red staining

At days 14 and 28, discs were rinsed twice in 1x PBS and fixed in 10% neutral buffered formalin for mineralization analysis via the alizarin red stain. Following fixation, discs were rinsed twice with deionized water and stained with alizarin red stock solution consisting of 1% wt/vol alizarin red in H2O with an adjusted pH of 4.2. Following staining, the samples were washed and destained with 10% cetylpyridinium chloride (CPC) for 15 minutes. 200uL of solution was transferred to 96 well plate and read at 562 nm using a SPARK® Multimode Microplate Reader (Tecan). Respective alizarin red staining was calculated from absorbance values and standard curve.

### Alkaline phosphatase assay

Conditioned media was collected at days 3, 7, 14, and 28 for quantification of alkaline phosphatase (ALP) over time. After all samples were collected and frozen, they were thawed for ALP analysis using a commercially available kit (ab83369, ABCAM). Undiluted samples (80 μl) and reagents were added to a 96 well plate per manufacturer’s instruction, plates were incubated for 1 hour, then stop solution was added to each well. Plate was shaken for 90 seconds and absorbance was measured at 405 nm using a SPARK® Multimode Microplate Reader (Tecan). ALP activity was calculated from absorbance values and standard curve using manufacturer’s instructions.

### L-lactate assay

*L*-lactate concentration was quantified in conditioned media collected at days 3, 7, 14, and 28 using a commercially available assay kit (MAK329, Sigma-Aldrich). Briefly, conditioned media samples (20 μl) were thawed to room temperature and added to respective wells of a clear, flat bottom 96 well plate. Separately, a reaction mix was prepared for each sample by mixing 60 μl Assay Buffer (MAK329A, Sigma-Aldrich), 1 μl Enzyme A (MAK329B, Sigma-Aldrich), 1 μl Enzyme B (MAK329C, Sigma-Aldrich), 10 μl NAD Solution (MAK329D, Sigma-Aldrich), and 14 μl MTT Solution (MAK329E, Sigma-Aldrich). 80 μl of Reaction Mix per sample was added to each well and mixed thoroughly. Immediately, the initial absorbance was measured at 565 nm (A_565_). The plate was allowed to incubate at room temperature for 20 min and the final absorbance was read at 565 nm (A_565_). A standard curve was generated according to manufacturer’s instructions. The initial A_565_ was subtracted from the final A_565_. Using the ΔA_565_ values, sample *L*-lactate concentration was quantified from the standard curve.

### Reverse-transcription quantitative polymerase chain reaction

Gene expression was analyzed at days 1 and 28 using primers to quantify osteoblast mRNA expression of *BGLAP*, *COL1A1*, and *BMP7* (**Table 3**).

**Table 3:** Human primers used for mRNA analysis.

Genes	Primer	Sequence (5'-3')
*Bmp7*	Forward Primer	TCACAGCCACCAGCAACCACTG
	Reverse Primer	ACCATGAAGGGCTGCTTGTTCT
*Bglap*	Forward Primer	CGCCTGGGTCTCTTCACTAC
	Reverse Primer	CTCACACTCCTCGCCCTATT
*Col1a1*	Forward Primer	GAGGGCCAAGACGAAGACATC
	Reverse Primer	CAGATCACGTCATCGCACAAC
*Gapdh*	Forward Primer	GTCTCCTCTGACTTCAACAGCG
	Reverse Primer	ACCACCCTGTTGCTGTAGCCAA

RNA was extracted using a commercially available kit (74004, Qiagen) according to manufactures instructions. The purity of the RNA was measured and quantitated on a Nanodrop-1000 spectrophotometer (Thermo Scientific, Wilmington, DE). iScript cDNA Synthesis Kit (Bio-Rad Laboratories) was used for reverse transcription of 1050 ng of total RNA. RT-qPCR was performed using SsoAdvanced Universal SYBR Green Supermix (Bio-Rad Laboratories) according to manufacturer’s instructions. Three technical replicates per sample were amplified using a CFX96 Real-Time System Thermal Cycler (Bio-Rad Laboratories). The gene target specificity of the reactions was evaluated with a melt curve generated at the end of the PCR amplification cycle. The average of the triplicates was used in calculations and samples that did not contain enough RNA to be run in triplicate were excluded from analysis. Relative gene expression compared to reference gene, *GAPDH*, was calculated from quantitative cycles (Cq) using the delta-delta Ct method:


$$\text{Relative RNA Expression}=2^{-(\text{ΔΔCt})}=2^{-(\text{ΔCt}(target)\;-\text{ ΔCt}(\text{GAPDH}))}$$

### Statistical analysis

Statistical analysis was performed using GraphPad Prism (version 6.01) software (GraphPad Software). The age and blood glucose levels between the patients from whom the healthy or diabetic osteoblasts were isolated from were compared using an unpaired student’s t-test, and the sex between groups was compared using chi-squared test. A two-way ANOVA was used to compare Alizarin Red concentrations, ALP activity, L-lactate concentrations and gene expression levels between high and low glucose concentrations in both the healthy and diabetic osteoblast groups. Data is reported as mean +/- standard error of the mean (SEM). Statistical significance was set at p<.05.

## Results

**Figure 2:**
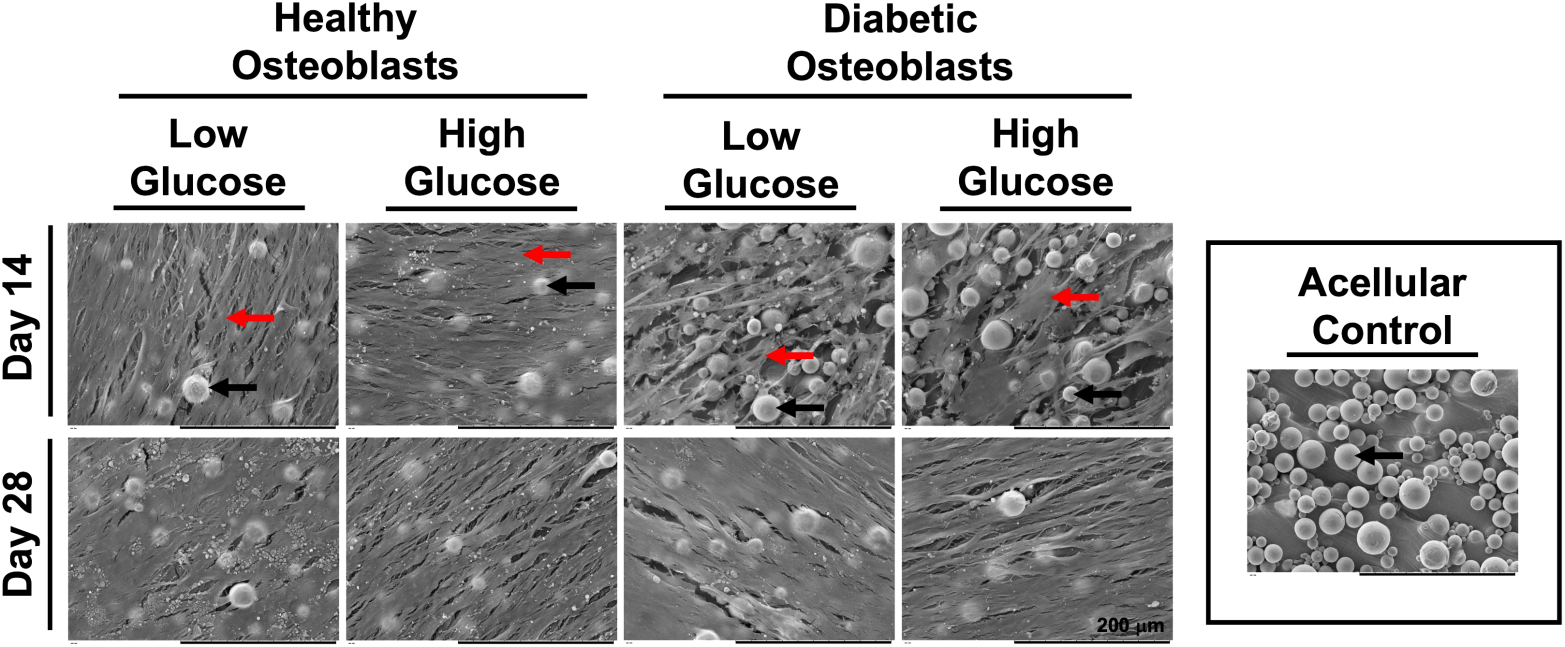
SEM micrographs of healthy and diabetic osteoblast groups cultured in high or low glucose medium at days 14 and 28. Acellular control disc is visualized in the outlined box on the right side of the figure. Osteoblasts and corresponding monolayers formed on the surface of the 3D fabricated discs are indicated using the red arrows. Fused spherical units of medical grade titanium powder (Ti6Al4V) encompassing the surface of the discs fabricated via L-PBF are identified by corresponding black arrows.

SEM images depicted in **Figure 2** reflect the cellular morphology and extracellular matrix (ECM) deposition of healthy and diabetic osteoblasts cultured in high and low glucose at days 14 and 28. At day 14, a dense and organized ECM can be observed in the healthy group, regardless of glucose concentration. Conversely, in the diabetic osteoblast group, much of the titanium surface (round blobs) is still visible and a less confluent deposition of ECM can be observed. At day 28, both healthy and diabetic osteoblast groups appear to maintain a dense and confluent ECM regardless of media glucose content.

**Figure 3:**
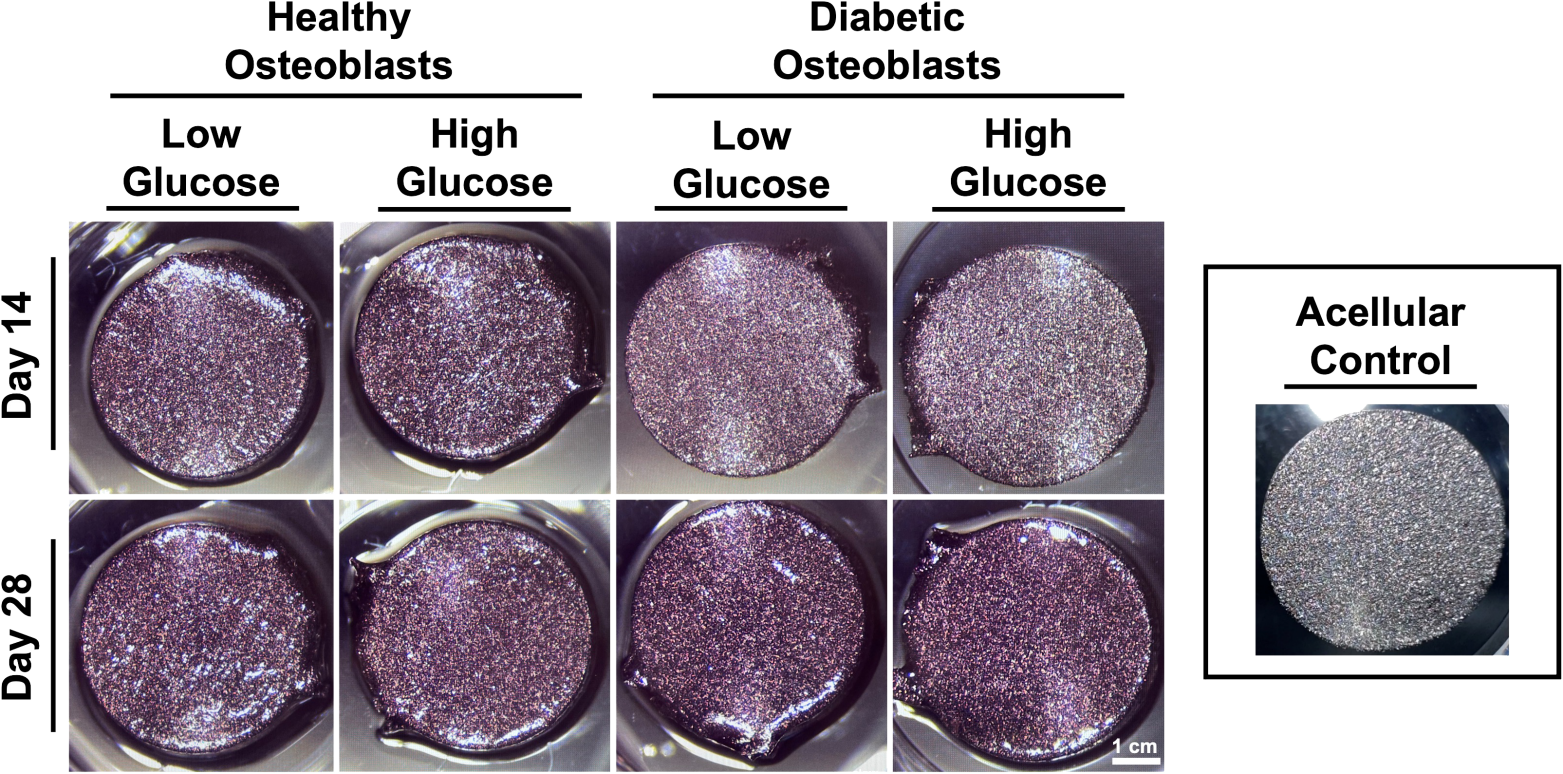
Representative gross images of osteoblast mineralization on titanium discs visualized by alizarin red staining.

**Figure 4:**
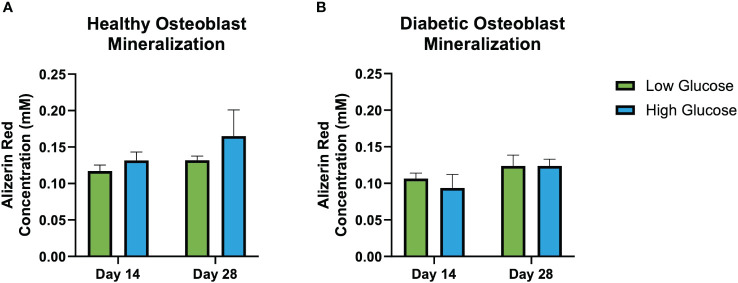
Alizarin red mineralization quantification. Healthy osteoblast alizarin red concentration (nM) **(A)**. Diabetic osteoblast alizarin red concentration (nM) **(B)**. Error bars represent SEM.

**Figure 3** shows Alizarin red staining reflecting mineralization at days 14 and 28. Quantification indicated a non-significant trend of increased mineralization in healthy osteoblasts cultured in both low and high glucose, and in diabetic osteoblasts cultured in higher glucose concentrations (**Figure 4**).

**Figure 5:**
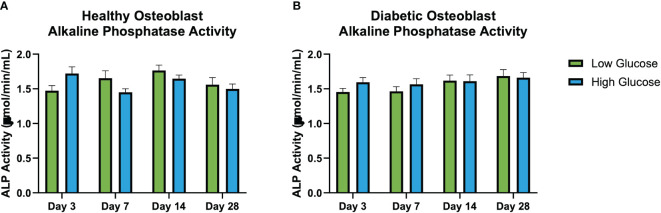
Illustrates the interaction between treatment and time on *in vitro* alkaline phosphatase activity (μmol/min/mL) (ALP) activity at days 3, 7, 14, and 28 in healthy osteoblasts **(A)** and diabetic osteoblasts **(B)**. Each cluster of bars represents different treatments administered at specific time points. The variation across the time points highlights the interaction effects, indicating if the effect of exposure to high of low glucose differs over time. Notably, **(A)** shows a significant interaction between treatment and time for healthy osteoblast ALP demonstrating the temporal dynamics of treatment responses not seen in the diabetic osteoblasts **(B)**. Error bars represent SEM.

No significant differences in alkaline phosphatase activity quantified from the conditioned medium at days 3, 7, 14, and 21 was observed (**Figure 5**). Regardless of the glucose concentrations of respective environments, the ALP activity of both diabetic and healthy osteoblasts remained conserved when compared at each timepoint. However, using 2-factor ANOVA (treatment, time) we see that there is a significant treatment vs time interaction in the healthy osteoblast group (p=0.04), indicating that the effect of treatment is time selective. There was no significant interaction between treatment and time in the diabetic group.

**Figure 6:**
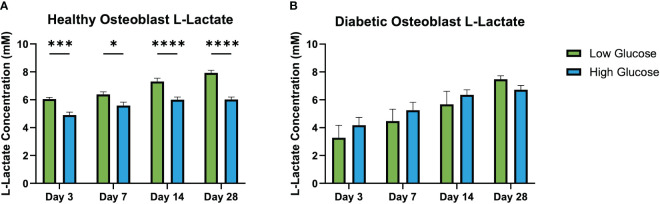
L-lactate concentration in conditioned medium at days 3, 7, 14, and 28. Healthy osteoblast L-lactate concentrations (mM) **(A)**. Diabetic osteoblast L-lactate concentrations (mM) **(B)**. Error bars represent SEM, *p<.05, ***p<.001, ****p<.0001.

Results of L-Lactate assay (**Figure 6**) found significantly higher levels of L-Lactate released by health osteoblasts cultured with low glucose medium compared to those cultured in high glucose medium. This difference was present at all culture time points (day 3, 7, 14 and 28). In the diabetic osteoblast group, there was no differences observed between those cultured in low or high glucose conditions at any time point.

**Figure 7:**
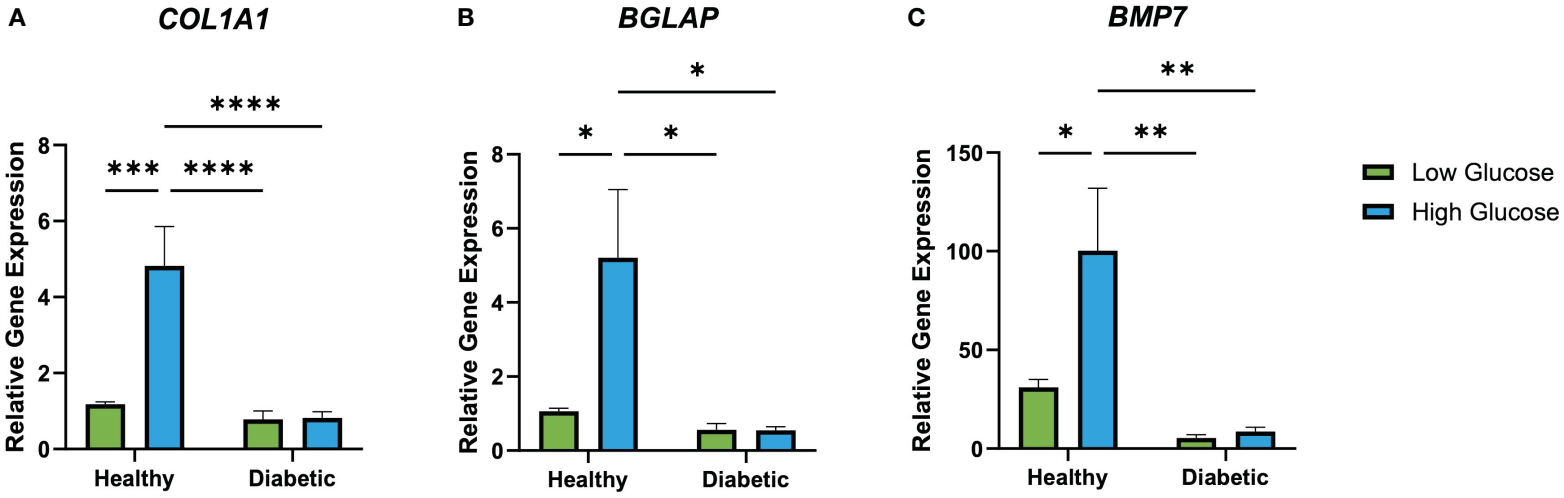
Relative healthy and diabetic osteoblast gene expression of *BGLAP* **(A)**, *COL1A1* **(B)**, and *BMP7* **(C)** measured at day 28 in respective culture conditions. Error bars represent SEM, *p<.05, **p<.01, ***p<.001, ****p<.0001.

RT-qPCR results (**Figure 7**) revealed an increased expression of *BGLAP* (osteocalcin) in healthy osteoblasts cultured in high glucose medium. While a significant increase in osteocalcin gene expression can be observed in healthy osteoblasts cultured in a high glucose environment, the expression levels of diabetic osteoblasts cultured in a high or low glucose environment remained unchanged. *COL1A1* expression was significantly elevated in healthy osteoblasts cultured in high glucose medium in comparison to the low glucose condition. Additionally, diabetic osteoblast *COL1A1* expression was unaffected when cultured in high or low glucose media. *BMP7* expression at day 28 was significantly higher in healthy osteoblasts cultured in high glucose medium in comparison to its low glucose counterpart. Furthermore, the same trend of increased high glucose healthy osteoblast *BMP7* expression can be observed with unchanging expression of diabetic osteoblasts cultured in high and low glucose environments.

## Discussion

Our investigation provides further insights into the influence of glucose environment on the behavior of osteoblast integration into 3D-printed titanium implants, which has potential clinical implications for post-surgery glucose management in patients with diabetes. It is well documented that diabetes can negatively impact bone healing following surgery through impaired osteoblast function and increased risk of nonunion (27–29). Nevertheless, understanding how diabetic osteoblasts respond to varying glucose environments in the context of a 3D-printed implant surface is paramount to guide patient management and optimize outcomes. Moreover, it is important to understand the diabetic osteoblast response in both basal and elevated glucose conditions.

While there was no difference among groups in ALP levels at individual timepoints, there was a significant interaction (treatment, time) in ALP levels in healthy osteoblasts in high and low glucose environments over time that was not observed in the diabetic group. There was no significant difference in mineralization activity in healthy or diabetic osteoblasts on 3D printed titanium discs when cultured in low or high glucose environment. Existing studies on diabetic osteoblast responses to changes in glucose are limited, and the current literature reports a wide range of responses in healthy cells in both mineralization and ALP activity in different glucose environments. For instance, Takeno et al. found an increase in mineralization in high glucose environments over a period of 21 days and an increase in ALP in the first 7 days (30). On the other hand, further studies report an increase in both mineralization and ALP activity in mildly increased glucose environments, but a decrease in mineralization and ALP activity when osteoblasts are exposed to high levels of glucose similar to the one used in this study (31–33). Meanwhile, a recent review looked at the combined effect of hyperglycemic conditions on ALP activity across multiple different studies and found overall no difference in response (34). However, it is important to note that these studies were performed using MC3T3 cell lines in culture and thus may account for the differences seen from the primary human osteoblasts cultured on 3D-printed titanium used in the present study. Also, the present study measured ALP activity from the conditioned media opposed to cell lysate to allow for temporal analysis which may have resulted in lower detection of its activity.

Additionally, the L-lactate assays conducted in this study provided further insights into the metabolic state of the osteoblasts under various glucose conditions. Higher concentrations of L-lactate in the media of healthy osteoblasts cultured in low glucose conditions could reflect an increased reliance on anaerobic glycolysis due to limited glucose availability (34). This shift towards anaerobic metabolism is a compensatory mechanism that enables cells to maintain energy production under glucose-restricted conditions (33). In contrast, the diabetic osteoblasts did not exhibit significant variations in L-lactate production between the high and low glucose environments, suggesting a potential metabolic inflexibility or an impaired metabolic response to glucose levels (35). These observations provide evidence of altered metabolic processes in diabetic osteoblasts, which may have implications for their function and the overall pathophysiology of diabetic bone disease (36). The metabolic flexibility in osteoblasts is crucial for bone health and may be detrimentally affected by diabetic conditions, challenging the effectiveness of postoperative glucose management in diabetic osseointegration (25). These results underscore the complex interplay between glucose levels and cellular metabolism in osteoblasts, further complicating the understanding of diabetic bone disease and the potential for intervention through glucose management.

The current literature on osteogenic gene expression in osteoblasts exposed to different glucose concentrations also contradicts. Zayazafoon et al. found healthy osteoblasts exposed to increased concentrations of glucose exhibited an increase in collagen 1 expression and decrease in osteocalcin (37), while other studies found an increase in osteocalcin after high glucose exposure (32, 38). At present there are no reports on *BMP7* gene expression in high and low glucose environments, however, *BMP7* has been shown to increase insulin secretion and glucose uptake in healthy individuals (39). Our data suggests a high glucose environment leads to increased osteogenic gene expression of *BGLAP*, *COL1A1*, and *BMP7* in healthy osteoblasts cultured on 3D-printed titanium discs. Overall, this finding is consistent with literature that has demonstrated that high glucose environments can stimulate osteoblast activity in non-diabetic conditions (40, 41). Osteoblasts mainly rely on glucose as their primary source of energy for bone remodeling and maturation and healthy osteoblasts have the ability to uptake and utilize increased surrounding glucose (34). Therefore, it follows that increasing glucose levels allows them to augment their energy stores and increase metabolic processes resulting in higher transcriptional activity of osteoblastic genes. Despite the upregulation of *BMP7*, we did not observe the anticipated increase in bone matrix production or significant changes in mineralization, as evaluated by Alizarin red staining and SEM. This discrepancy suggests that in a high glucose environment, other regulatory mechanisms may modulate *BMP7*’s osteoinductive effects, or the role of *BMP7* in matrix production and mineralization could be inherently more intricate than previously recognized.

In contrast to the healthy osteoblasts, we did not observe any significant changes in osteogenic gene expression in the diabetic group under high glucose conditions. This data further emphasizes that diabetic osteoblasts are largely unresponsive to variations in glucose levels. This lack of response in the diabetic group is in line with studies reporting reduced osteoblast activity and responsiveness in diabetic conditions, which have been attributed to insulin resistance and oxidative stress (17, 35). This finding corroborates that there may be an inherent dysfunction in diabetic osteoblasts that prevents them from utilizing available glucose in the same manner as a healthy cell (36).

Overall, the lack of mineralization, proliferation and gene expression response in the diabetic group in high glucose environments suggests that the metabolism and maturation osteoblasts of diabetic patients are unchanged by an increase in available glucose. The significant response to increased glucose in healthy osteoblasts, while different than some of the published literature, is unsurprising given the increase in readily available source of energy for these normally functioning cells. Therefore, the lack of response in the diabetic osteoblasts may inherently suggest that they do not have typical bone building potential regardless of their glucose environment.

Our findings call into question the current clinical guidelines which advocate for strict glycemic control following orthopedic surgeries to reduce complications related to bone healing (42). Instead, our results suggest that the osteogenic potential of diabetic osteoblasts may not be detrimentally affected by high glucose levels, at least in the context of osseointegration of 3D-printed titanium implants. This has potential implications for the management of postoperative hyperglycemia in patients with diabetes, suggesting that aggressive glucose control may not be necessary to ensure optimal osseointegration. However, we must consider that systemic effects of uncontrolled blood glucose such as increased risk of infection or cardiovascular events could still warrant strict glucose management following surgery (43, 44).

These findings, however, must be interpreted within the limitations of our study. Our *in vitro* model cannot fully replicate the complex physiological environment within a living organism. The human body presents a myriad of other factors, including inflammatory responses, hormonal changes, and vascular supply, all of which have significant influences on osteoblast function and bone healing that were not accounted for in our study. Additionally, in this study we used cells derived from a limited number of individuals, and inter-individual variability might affect the response to different glucose concentrations. There was also an observed age difference between patients from which the healthy and diabetic osteoblasts were derived. While this difference was not significant, it is important to recognize that younger osteoblasts may exhibit higher activity levels at baseline. Also, in the present study the ALP levels were measured from the conditioned media as opposed to cell lysate so we could observe temporal changes over the course of the experiment. Even though we found significant variation in responsiveness in healthy osteoblasts in high and low glucose environments over time, we may not have captured the whole picture. While ALP activity in the conditioned media can indicate the secretion of the enzyme by the osteoblasts, it may not fully reflect the intracellular level of ALP within those cells. Despite these limitations, our study offers new insights into the influence of glucose environment on osteoblast behavior in the context of 3D-printed titanium implants. Future research should validate our findings in larger, more diverse cohorts and *in vivo* models, which may provide more physiological relevance.

## Conclusion

In this study we characterize the impact that a high or low glucose environment plays on primary diabetic and healthy osteoblasts to contribute to the understanding of how glucose levels may impact the integration of 3D-printed implants in patients with diabetes. Our findings suggests that diabetic osteoblasts on a 3D-printed titanium surface exhibit diminished responsiveness to glucose variations compared to healthy cells, which has potential implications for post-surgery glucose management strategies. Further research is warranted to confirm these findings and explore potential mechanisms underlying the observed osteoblast dysfunction in diabetes to guide treatment decisions and postoperative recommendations.

## Data availability statement

The raw data supporting the conclusions of this article will be made available by the authors, without undue reservation.

## Ethics statement

The studies involving humans were approved by the Duke University Health System Institutional Review Board (IRB) (protocol number Pro00033747). The studies were conducted in accordance with the local legislation and institutional requirements. The ethics committee/institutional review board waived the requirement of written informed consent for participation from the participants or the participants’ legal guardians/next of kin because the IRB determined the protocol meets the definition of research not involving human subjects.

## Author contributions

NA: Conceptualization, Data curation, Investigation, Methodology, Visualization, Writing – review & editing. AA: Formal analysis, Visualization, Writing – original draft, Writing – review & editing. BA: Data curation, Investigation, Visualization, Writing – review & editing. JB: Data curation, Investigation, Writing – review & editing. MM: Data curation, Writing – review & editing. LD: Data curation, Writing – review & editing. RD: Data curation, Investigation, Writing – review & editing. SA: Conceptualization, Data curation, Formal analysis, Investigation, Methodology, Resources, Supervision, Writing – original draft, Writing – review & editing.
